# The Clinical Significance and Application of Heart Rate Variability in Dialysis Patients: A Narrative Review

**DOI:** 10.3390/biomedicines12071547

**Published:** 2024-07-11

**Authors:** Rong-Na Jhen, Ping-Chen Wang, Yu-Ming Chang, Jsun-Liang Kao, Eric Chien-Hwa Wu, Chih-Chung Shiao

**Affiliations:** 1 Division of Nephrology, Department of Internal Medicine, Camillian Saint Mary’s Hospital Luodong, No. 160, Zhongzheng S. Rd., Luodong Township, Yilan County 265, Taiwan; wynnazhen@gmail.com (R.-N.J.); ynk123.tw@yahoo.com.tw (Y.-M.C.); smh01068@smh.org.tw (J.-L.K.); 2 Department of Medical Research and Education, Camillian Saint Mary’s Hospital Luodong, No. 160, Zhongzheng S. Rd., Luodong Township, Yilan County 265, Taiwan; pw279@outlook.com; 3 Division of Nephrology, Department of Internal Medicine, Camillian Saint Mary’s Hospital Jiaoxi, No. 129, Sec. 4, Jiaoxi Rd., Jiaoxi Township, Yilan County 262, Taiwan; planetarium0527@gmail.com

**Keywords:** autonomic nervous system, end-stage kidney disease, heart rate variability, dialysis, prediction, prognosis

## Abstract

Autonomic nervous system (ANS) dysfunction is prevalent in end-stage kidney disease (ESKD) patients, carrying significant risks for morbidity and mortality. Heart rate variability (HRV) is a simple and non-invasive method to evaluate ANS functions and predict prognoses in specific patient populations. Since there is a lack of a clear understanding of the clinical significance of HRV in predicting prognoses in ESKD patients, an updated review on this topic is urgently warranted. The clinical significance of HRV in dialysis patients includes its associations with metabolic syndrome, nutritional status, intradialytic hypotension, vascular access failure, major adverse cardiovascular events, and mortality. These findings underscore the essential role of the autonomic reserve, which might denote the elevation of ANS activity as a response to external stimulus. Patients with a higher level of sympathetic activity at the resting stage, but who are unable to adequately elevate their sympathetic activity under stress might be susceptible to a worse outcome in critical circumstances. Further applications of HRV include HRV biofeedback, risk classification, and real-time HRV monitoring. Overall, HRV is an optimal tool for predicting prognoses in dialysis patients. Further study is encouraged in order to gain a clearer understanding of the clinical significance and application of HRV, and thereby enhance the care of ESKD patients.

## 1. Introduction

Cardiovascular disease is one of the leading causes of death in patients with chronic kidney disease (CKD), including end-stage kidney disease (ESKD) necessitating hemodialysis (HD) or peritoneal dialysis (PD). Autonomic nervous system (ANS) dysfunction, observed in over half of all chronic dialysis patients [1], has emerged as one of the main mechanisms underlying sudden cardiac death in ESKD patients. ANS dysfunction induces a reduced end-organ response of circulating catecholamines, disturbances in cardiac function, and derangements in the sympathetic and parasympathetic nervous system in CKD patients [2], presenting clinical manifestations including hypertension, orthostatic hypotension, intradialytic hypotension, resting tachycardia with fixed heart rate, or abnormal heart rate variability (HRV) [2,3,4,5]. Thus, the application of HRV measurement can also provide insights regarding autonomic neuropathy-related symptoms and further predict the patients’ outcomes [6,7].

**Figure 1:**
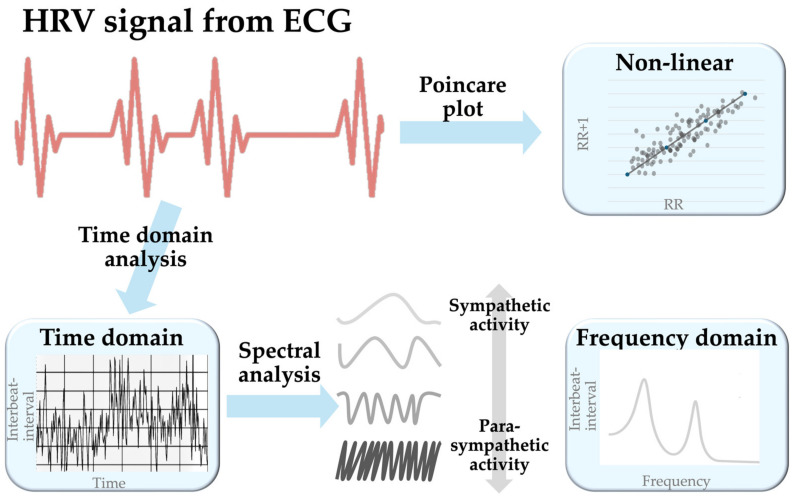
A schematic diagram showing the generation of the three HRV models. Abbreviation: ECG, electrocardiography.

HRV represents the oscillation between consecutive heartbeats captured with electrocardiography (ECG). HRV detection involves multiple steps to measure the R-R interval from the QRS complex. After obtaining the raw ECG signal, a preprocessing step filters and removes noise to make it smoother and cleaner for QRS complex detection. In the next step, robust algorithms like Pan–Tompkins, wavelet transform, and empirical mode decomposition aid in detecting and statistically evaluating R-R intervals to generate the time domain model. Subsequently, the spectral transformation of the time domain model through methods like Fast Fourier Transform, autoregressive models, the Welch periodogram, and the Lomb–Scargle periodogram generates the frequency domain model [8,9]. During the transformation to the non-linear model, the introduced methods may incorporate approximate entropy, Poincare plots (a type of recurrence plots named after Henri Poincaré), recurrence plots, detrended fluctuation analysis (DFA), or the correlation dimension [10]. Among these methods, the Poincare plot is widely accepted [11] (Figure 1).

Clinically, HRV measurement is a simple and non-invasive method used to evaluate ANS functions and predict specific morbidity and mortality in certain patient populations [12]. HRV measurements were initially employed to predict the mortality rates in populations with ischemic heart or cardiovascular diseases (CVD) [13], and subsequently extended to the prediction of cardiovascular and all-cause morbidity and mortality in many clinical settings, including those involving ESKD patients [14]. Many studies have demonstrated associations between HRV and various clinical statuses and illnesses in ESKD patients [6,15,16,17,18,19,20,21,22]. However, among these studies, the time points of HRV measurement [e.g., before HD (pre-HD), during HD, or after HD (post-HD)], choices of HRV indices, and utility of HRV values (e.g., single values of HRV measurement, or the dynamic changes of specific HRV indices) vastly vary. The significant variation among these studies prohibits a clear understanding of the clinical significance of HRV in predicting prognoses in ESKD patients; thus, a review of the available data on HRV is urgently warranted. This narrative review updates and summarizes the current knowledge and indicates a potential direction for further research into this good clinical tool that can provide better care for ESKD patients.

## 2. Autonomic Imbalance and HRV in CKD Patients

Physiologically, receptors within the kidneys are activated and stimulated to send renal afferent signals to the central nervous system (CNS), triggering efferent sympathetic activity [23]. Meanwhile, sympathetic activity can also be stimulated by other factors such as insulin, endothelin, and sodium [24]. Increased sympathetic activation causes a rise in basal heart rate and promotes cardiac remodeling and fibrosis, which may worsen cardiac function [25]. Conversely, increased parasympathetic activity reduces reflex control of autonomic activity, nitric oxide bioavailability (central/peripheral), renalase, and atrial natriuretic peptide [26]. Activating a parasympathetic tone facilitates rest and digest perception while increasing performance, adaptability, and cognition [27]. Clinically, ANS imbalance represents a higher heart rate, reduced adaptability cognition, and ease of exhaustion [7].

HRV information can be interpreted using time domain, frequency domain, and non-linear models. In the time domain model, SDNN (the standard deviation of the normal-to-normal interval) indicates the total activity of sympathetic and parasympathetic components. In contrast, RMSSD (root mean square successive differences) and NN50 (number of pairs of adjacent NN intervals differing by more than 50 ms in the entire recording) are taken as an index for parasympathetic activity [28]. The frequency domain model includes several indices such as total power (TP), very low frequency (VLF), low frequency (LF), low frequency percentage (LF%), high frequency (HF), high frequency percentage (HF%), and LF/HF ratio (low-to-high frequency ratio) [28], as well as normalized LF (nLF) and normalized HF (nHF), which are introduced as a ratio presented in percentile units calculated by dividing LF and HF by TP, respectively [29]. Among these indices, TP is considered as all spectra of the frequencies; LF, LF%, and nLF activity indicate the sympathetic effects; HF, HF%, and nHF activity have been related to parasympathetic nervous activity, which represents the vagal-mediated modulation of heart rate; LF/HF ratio is an index of sympathetic to parasympathetic balance; and VLF is considered to reflect vasomotor function, the renin–angiotensin–aldosterone system, and the cardiac response toward external stress [29,30,31,32]. As for the third and least frequently used model, the non-linear model, SD1 (the standard deviation of instantaneous beat-to-beat interval variability) [33] and SD2 (the continuous long-term R-R interval variability) [33] indicate parasympathetic activity and total autonomic activity, respectively. (Table 1)

The measurement of HRV follows a particular time frame, with RMSSD, SD1, and SD2 being recorded for at least 5 min; LF and NN50 being recorded for a minimum of 2 min; HF being recorded for at least 1 min; and SDNN, VLF and LF/HF ratio can be captured with either 24 h or 5 min methods [12]. Generally speaking, long-term (with ECG of 1–24 h) HRV analysis is a stable tool for assessing ANS function, describing the ANS function change over hours, and reliably predicting patients’ prognoses, while short-term (with ECG of several minutes) HRV analysis acts as a convenient method for estimating autonomic status and tracking dynamic changes in cardiac ANS function within minutes [34]. Although the application of 24 h HRV recording is regarded as the gold standard, the 5 min HRV measurement is also considered an optimal tool that is less time consuming, and is widely accepted [12].

In patients with CKD, the impaired reflex control of autonomic activity, activation of the renin–angiotensin–aldosterone system, and structural remodeling of the heart [26] lead to ANS imbalance, including an over-activated sympathetic system and a reduced parasympathetic activity level [26]. The sympathetic nerve activity level increases progressively with a declining estimated glomerular filtration rate from normal renal function to advanced CKD needing HD support [35]. Evidence of sympathetic activation in CKD patients is bolstered by increased LF and LF/HF ratio and decreases in SDNN, HF, baroreceptor sensitivity, and renal perfusion [27].

## 3. Clinical Significance of HRV in Dialysis Patients

The clinical significance of HRV in dialysis patients has been demonstrated in association with metabolic syndrome (MetS) [16,17], nutritional status [15], intradialytic hypotension (IDH) [18,22], vascular accesses failure (VAF) [19], major adverse cardiovascular events (MACE) [20,21], and mortality [6,22] (Table 2).

### 3.1. HRV and MetS

The relationship between MetS and stress has been disclosed in many studies [16,17,43]. Stress is recognized as a significant factor driving ANS, potentially leading to lipid metabolism and generating insulin resistance and blood pressure (BP) [17]. At the same time, MetS is known as a cluster of atherosclerotic risk factors that consist of the interplay between the HRV indices [16,43]. This knowledge further outlines a potential link between stress-induced ANS activation and the development of MetS.

Given the research gap regarding the relationship between HRV and MetS in chronic HD patients, Chang et al. delivered a cross-sectional study recruiting 175 HD patients (mean age 65.1 years, women 57.1%) [16]. The authors evaluated the association between MetS and the four 5 min HRV measurements, including one before HD and three during the index HD. The study revealed that patients with MetS had significantly lower values of several HRV indices. Further subsequent analysis found that the fasting plasma glucose (FPG) criterion significantly influenced most HRV indices (including those representing total autonomic power, as well as sympathetic and parasympathetic activities). In contrast, the other four components, including “BP”, “waist circumference”, “high-density lipoprotein cholesterol”, and “triglycerides” criteria, showed little impact on HRV. Notably, the FPG criterion had the most potent influence on cardiac ANS, even higher than that of MetS [16]. Both sympathetic and parasympathetic activities have been linked to insulin resistance and type 2 DM [44,45], indicating the critical role of abnormal glucose metabolism in ANS dysfunction. The study by Chang et al. further emphasized that the impact of FPG (+) on cardiac ANS is still pronounced even in situations with uremic autonomic dysfunction [16].

The association between MetS and ANS dysregulation in HD patients was supported by a community-based prospective study enrolling 1933 participants in a primary care setting that demonstrated that ANS dysregulation can predict the development of MetS [17]. Another study enrolling 1011 elderly participants without cardiovascular disease found that MetS and some components of MetS were independently associated with decreased long-term HRV values, while long-term (24 h and nighttime) HRV measurements performed better than short-term (5-min) HRV measurements in evaluating ANS control alteration [46].

### 3.2. HRV and Malnutrition

Using the same study population as Chang et al.’s work [16], Wu et al. conducted a cross-sectional study enrolling 175 HD patients (mean age 65.1 years, women 57.1%) to investigate the association between nutritional markers and four HRV measurements during the index HD [15]. By applying a multivariate generalized estimating equation with adjustment, the study found that the values of HRV indices were independently associated with malnutrition defined by serum albumin < 3.8 g/dL, total cholesterol < 100 mg/dL, body mass index < 23 kg/m^2^, body weight loss (>10% within six months, or >5% within three months), and normalized protein catabolic rate < 1.1 g/kg BW/d. This study demonstrated ANS impairment in HD patients with malnutrition. These findings further indicated that ANS dysfunction might be a potential mechanism linking malnutrition to subsequent adverse prognoses in HD [15].

### 3.3. HRV and Mortality

#### 3.3.1. Pre-HD HRV Predicts Mortality in HD Patients

To elucidate the significance of HRV in patient survival, Kuo et al. conducted a prospective cohort study enrolling 41 HD patients (mean age 59.5 years, women 48.7%) [36]. HRV was measured before the HD sessions using a 5 min ECG recording as a baseline. The study reported that 35.7% of patients died during the 150-month median follow-up period. A subsequent Cox proportional hazards model found that a higher LF/HF ratio [hazard ratio (HR) = 3.03, *p* = 0.03] during pre-HD HRV measurement and diabetes mellitus (DM) (HR = 3.49, *p* = 0.02) were independent predictors of all-cause mortality.

#### 3.3.2. HRV during HD Predicts Mortality in HD Patients

Another study by Polikakos et al. [37] enrolled 72 HD patients (mean age 61.0 years, women 31.9%) to investigate the relationship between HRV during HD and patients’ outcomes within a median follow-up period of 54.8 months. The study found that a lower LF/HF ratio during the first hour of HD was an independent predictor for all-cause mortality.

#### 3.3.3. Post-HD HRV Predicts Mortality in HD Patients

Subsequently, Osataphan et al. conducted a prospective cohort study enrolling 163 HD patients (mean age 61.4 years, women 46.0%) to evaluate the ability of HRV to predict patient survival [6]. The HRV was measured using a Holter ECG recording that began ten minutes before HD, continued through the 4 h HD session, and ended ten minutes after HD. The study reported a mortality rate of 22.7% within the median follow-up period of 40 months and demonstrated that the HRV indices measured at the post-HD stage, but not at the pre-HD or during HD stages, independently predicted all-cause mortality. These independent predictors from post-HD HRV measurement included lower VLF (HR = 0.88, *p* < 0.001), lower nLF (HR = 0.95, *p* = 0.005), lower LF/HF ratio (HR = 0.23, *p* = 0.004), and higher nHF (HR = 1.05, *p* = 0.005). Among these predictors, VLF showed the best predictive ability, presenting the highest area under the receiver operating characteristic curve (AuROC = 0.71, *p* < 0.001) for survival compared to the other post-HD HRV indices [6].

#### 3.3.4. Change in HRV during HD Predicts Mortality in HD Patients

Chen et al. provided a different view of predicting mortality in HD patients through the HRV (ΔHRV) change, defined as the difference in HRV values between the post-HD and pre-HD stages [38]. This study included 182 HD patients (mean age 61.2 years, women 55.5%) and followed them, with a median follow-up period of 35.2 months, to examine ΔHRV’s ability to predict overall and CV mortality in HD patients. During the follow-up period, the deaths of 15.9% of the cohort occurred. The study found that the VLF, LF%, and LF/HF ratio increased in the post-HD HRV measurement in survived patients, but not in those who died. The patients with ΔLF% higher than the median value (5.1 nu) were found to have higher survival rates. The multivariate analysis disclosed that decreased ΔLF% was associated with increased overall (HR = 0.98, *p* = 0.02) and cardiovascular mortality (HR = 0.94, *p* < 0.001), which were more potent than in the pre-HD HRV measurement. Furthermore, adding ΔLF% to a clinical model increased the model’s predictive ability for all-cause mortality (*p* = 0.002) and cardiovascular mortality (*p* < 0.001) [38].

#### 3.3.5. Repeated HRV Measurements Predict Mortality in HD Patients

The dynamic change of HRV indices during the HD process might be a more relevant predictor than a single HRV measurement in predicting patient outcomes. Chang et al. conducted a study using a prospective cohort of stable HD patients to test the predictive ability of repeated HRV measurements (one before and three during HD) for long-term patient prognoses [7]. Among the 164 enrolled patients (mean age 65.0 years; women 57.3%), 48.2% died within the eight years of the follow-up period. The study utilized a multivariate mixed model to calculate the adjusted beta coefficients of the individual HRV indices and the joint modeling method to evaluate the independent predictors among the HRV indices after adjusting the independent risk factors found in the multivariate Cox regression. Finally, the study demonstrated that higher nHF (HR = 1.03), lower VLF (HR = 0.99), lower variance (HR = 0.99), nLF (HR = 0.99, *p* = 0.006), and LF/HF ratio (HR = 0.8) were independent predictors for cardiovascular mortality. Meanwhile, higher nHF (HR = 1.03) was an independent predictor for infection-associated mortality (all *p* < 0.001 unless otherwise addressed). The authors thus concluded that repeat HRV measurements can predict long-term mortality among HD patients [7].

#### 3.3.6. The HRV Indices with Clinical Significance in HD Patients

Since the results regarding HRV in HD patients remain inconsistent, Yang et al. conducted a meta-analysis to evaluate the association between HRV measurement and mortality in HD patients and the most reliable HRV indices for such prediction [14]. The meta-analysis of seven eligible studies found that decreased HRV was associated with higher all-cause mortality (HR = 1.63, *p* = 0.01) and cardiovascular mortality (HR = 1.07, *p* = 0.045). Among the various HRV indices, decreased SDANN (standard deviation of the average NN intervals) (HR = 1.02, *p* < 0.001) and decreased LF/HF ratio (HR = 2.81, *p* = 0.001) were disclosed as predictors of all-cause mortality, whereas decreased SDNN (HR = 1.03, *p* = 0.004), SDANN (HR = 1.03, *p* = 0.001), LF/HF ratio (HR = 8.69, *p* = 0.002), and Ln LF (HR = 1.58, *p* < 0.001) were predictors of cardiovascular mortality. In addition, it is worth mentioning that the association with all-cause mortality was more significant in long-term ECG recordings than in short-term recordings [14].

#### 3.3.7. HRV Predicts Mortality in PD Patients

Aside from HD patients, the predictive ability of HRV for patient survival was also demonstrated in PD patients. In a prospective observational study enrolling 81 PD patients (mean age 65.3 years, women 49.4%), Pei et al. evaluated the association between HRV measures and mortality within the 43.8 months of the follow-up period [39]. The HRV was measured between 8 am and 10 am using a 5 min ECG when these PD patients were in adequate condition. Compared with survivors (*n* = 56), the non-survivors (*n* = 25) had significantly higher RMSSD (the square root of the mean of the squared differences between adjacent normal-to-normal intervals), SDSD (the standard deviation of differences between adjacent normal-to-normal intervals), and nHF, but lower nLF and LF/HF ratios. Using the multivariate Cox proportional hazards model, the authors found that a lower LF/HF ratio (RR = 0.70, *p* = 0.03), indicating impaired sympathetic nerve regulation is an independent predictor of all-cause mortality in PD patients [39].

Another study by Chiang et al. further consolidated the significance of HRV indices in PD patients by enrolling 132 PD patients (mean age 53.7 years, women 55%) [9]. The study found that a lower short-term DFA (DFAα1), which indicates decreased sympathetic activity and increased vagal activity, strongly predicted increased mortality. The study further determined that DFAα1 < 0.95 is an independent predictor for cardiac and all-cause mortality [9].

### 3.4. HRV and MACE

It is known that MACE is a leading cause of mortality and disability in end-stage renal disease patients [47]; thus, Kida et al. conducted a prospective observation study to evaluate the association between MACE and HRV, which was measured using a 24 h Holter ECG before the HD initiation of the patients [40]. Among the 90 analyzed HD patients (mean age 63.4 years, women 28.9%), 36.7% developed MACE during a mean follow-up period of 32 months. The study disclosed that SDNN and SDANN were significantly lower in patients with MACE than those without MACE. The Kaplan–Meier analysis showed a worse MACE-free survival rate in the patients with lower SDNN and SDANN. Furthermore, lower SDNN (HR = 0.57, *p* = 0.002) was proven as an independent predictor for MACE development. Nevertheless, the independent association between SDNN and MACE existed in non-diabetic patients, but not diabetic patients. The authors concluded that HRV measured by 24 h Holter ECG helps predict MACE in HD patients, especially the non-diabetic group.

Another study addressing the association between HRV and MACE in HD patients is a prospective cohort study enrolling 179 HD pts (mean age 61.2 years, women 55.3%) by Huang et al. [21]. HRV was measured before HD treatment using a 5 min ECG recording. During a mean follow-up period of 33.3 months, 20.1% of patients had a MACE and 54.7% experienced hospitalization. Kaplan–Meier curves found decreased MACE-free survival in the patient groups with decreased VLF levels. Finally, the multivariate-adjusted Cox regression analysis demonstrated that low VLF activity independently predicted higher risks of MACE (HR = 0.73, *p* < 0.001) and hospitalization (HR = 0.87, *p* = 0.012) in maintenance HD patients. The association between HRV and MACE has also been shown in other populations. In patients with acute coronary syndrome, HRV indices were identified as significant indicators for MACE occurrence [LF (AUC = 0.7 and 95% CI = 0.54 to 0.85) and LF/HF ratio (AUC = 0.85, 95% CI = 0.74 to 0.96)] [20]. Additionally, as mentioned earlier, the study by Polikakos et al. [37] also disclosed that a lower LF/HF ratio during the first hour of HD is an independent predictor for MACE.

### 3.5. HRV and IDH

IDH is a common complication during HD that precludes optimal ultrafiltration and clearance during HD and causes adverse impacts, including VAF, mortality, or other unexpected events in HD patients [18]. The prospective study by Chang et al. evaluated the association between IDH and HRV, measured using a 5 min ECG four times (one measured pre-HD and three measured during HD) in the index HD session [22]. After categorizing 171 enrolled patients (mean age 64.9 years, women 56.1%) into three groups according to their intradialytic changes in systolic BP, the authors found that the patients with IDH had statistically lower levels of many HRV indices from the middle phase of HD. By logistic regression analysis, a higher LF/HF ratio [odds ratio (OR) = 1.72, *p* = 0.02] and lower variance (OR = 0.64, *p* = 0.05) at HD initiation were independently associated with IDH.

In addition, Park et al. conducted a prospective study to test whether HRV could predict IDH one month in advance for HD patients [41]. The study enrolled 71 HD patients (mean age 54.8 years, women 43.7%), measured HRV by a 5 min ECG at early, middle, and late phases of the index HD, and then recorded IDH events during the following one-month observation period. The study found that the changes in many HRV indices between the early and middle phases of HD (ΔHRV) were among the independent predictors for IDH within the following month. Furthermore, the ΔHRV variables significantly improved the predictive performance of a multivariate model, which could predict IDH when added to (*p* = 0.05).

Recently, Park et al. [42] conducted a multicenter prospective observational study recruiting 70 HD patients (44.3% ≥ 65 years, women 50%) to evaluate the association between HRV in the non-HD phase and IDH. After demonstrating the association between HRV indices and IDH, the authors proposed the HRV-IDH index (including LF, LF/HF ratio, VLF, TP, and NN50) and proved a robust correlation between this index and IDH development. The authors finally concluded that ANS dysfunction, determined by the HRV-IDH index, is an independent risk factor for IDH.

### 3.6. HRV and VAF

Vascular access (VA) is considered a lifeline of HD patients; thus, Huang et al. conducted a retrospective study analyzing a prospectively established cohort with 175 chronic HD patients (mean age 65.1 years, women 57.1%) to evaluate the predictive values of the HRV indices for long-term VA outcomes [19]. During the 60-month follow-up period, 26.8% of patients experienced VAF events. The values of most HRV indices were statistically increased during HD since initiation in the patients without VAF, but not in those with VAF. In all participants, higher nHF activity (HR = 1.04, *p* = 0.005) and a lower LF/HF ratio (HR = 0.80, *p* = 0.015) were two independent indicators for VAF. Further subgroup analysis disclosed that a lower sympathetic activity level indicated by a lower LF/HF ratio was an independent indicator for VAF (HR = 0.61, *p* = 0.03) when using a tunneled cuffed catheter, but it conversely played a protective role against VAF (HR = 1.27, *p* = 0.002) for arteriovenous fistula.

## 4. Discussion

### 4.1. Current Clinical Significance

The ANS contains sympathetic and parasympathetic activities, which act in a “growth and decline” fashion. The ANS promptly responds to various stimuli to maintain human vital functions [48]. The sympathetic activity gradually increases following the worsening renal function from the early stage [49], but tends to decrease after receiving HD treatment [18]. In ESKD patients, cardiac ANS abnormality is characterized by an overall decreased HRV, overactive sympathetic tone, and reduced parasympathetic tone [18,50,51]. When patients face stress generated during HD, the initial compensatory stage results in an activated sympathetic tone with increased general HRV values, indicative of an adequate autonomic reserve and better patient outcomes [36]. However, when the sympathetic stimulation is too intense or prolonged, sympathetic withdrawal might occur, denoting an inadequate response that predisposes patients to worse outcomes [18,19,52,53]

This updated review summarizes the clinical significance of HRV in dialysis patients. Higher FPG—a vital component of MetS—is associated with decreased HRV, denoting decreased autonomic, sympathetic, and parasympathetic activities [16]. Also, the malnutrition status defined by the criteria of protein-energy wasting syndrome is associated with ANS dysfunction [15]. Existing data show that a higher sympathetic activity level before HD [36], a lower sympathetic activity level after HD [6], an inability to raise sympathetic activity during HD [38], and an overall lower sympathetic activity level during HD [7] are associated with higher mortality risks in HD patients. In addition, lower sympathetic activity has also been associated with a higher mortality rate for PD patients [39]. Regarding MACE, lower ANS activity [40] and a lower cardiac response toward external stress at the pre-HD stage [21] are associated with higher MACE risks. As for IDH, a lower sympathetic activity level at early stages of HD can predict a higher risk of IDH at the index HD [22], while a lower increase in ANS activity during HD has been associated with a higher IDH risk within the coming month [41] (Table 3).

Taking the above information together, we underscore the essential role of the autonomic reserve, which might denote the elevation of ANS activity, including its sympathetic and parasympathetic aspects, as a response to external stimuli [7,22]. Patients with a higher sympathetic activity level and relatively lower parasympathetic activity at the resting stage, but who are unable to adequately elevate their sympathetic activity level under stress might be susceptible to a worse outcome in critical circumstances [7].

### 4.2. Other Factors Associated with HRV and Patients’ Outcomes

The impact of DM on HRV is also worth noting. Various studies have positioned their aims based on this domain [54], while some research defines it as an attribute among their participants [36]. Such a role might mediate between HRV and the targeted significance. For example, an association between SDNN and HRV was found in the research of Kida et al., while in patients with DM, the relationship did not show statistical significance [40]. Interestingly, a study by Park et al. found that in a cohort of HD patients with IDH, there were more patients with DM compared to a cohort of non-IDH patients [42]. However, this association might be ambiguous and need further confirmation. The factor of DM might, to some extent, affect HRV indices, and future research is needed to extend the field of knowledge regarding the impact of DM on HRV measurement.

Controversial results exist regarding the influence of electrolytes on HRV in ESKD patients. The changes in sodium, potassium, chloride, calcium, phosphate, and bicarbonate concentrations during HD do not correlate with HRV [55,56,57]. In comparison, the magnesium concentration is negatively correlated with parasympathetic activity [56]. In addition, intradialytic exercise has also been reported to improve HRV indices [58], yet the field of study might seem to detour from the scope of the impact and clinical significance of HRV.

### 4.3. Future Application

**Figure 2:**
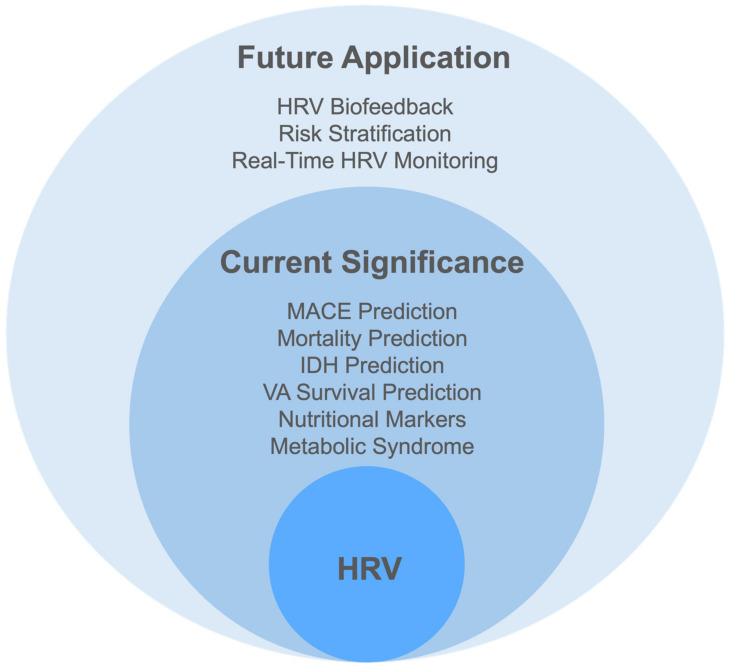
The clinical significance and application of heart rate variability. Abbreviations: HRV, heart rate variability; IDH, intradialytic hypotension; MACE, major adverse cardiovascular events; VA, vascular access.

HRV applications include HRV biofeedback, risk classification, and real-time HRV monitoring (Figure 2).

#### 4.3.1. HRV Biofeedback (HRVB)

HRV biofeedback is a technique to improve ANS function. Patients are trained to control their HRV patterns to increase the amplitude of respiratory sinus arrhythmia, which is measured by real-time feedback on HRV measures [59]. The ideal breathing resonant frequency should be 4.5 to 6 b/m [60]. This control over breathing is reported to stimulate the parasympathetic nervous system and mitigate the influence of sympathetic nervous system activation, which boosts the vagus nerve activity [59]. Consequently, a higher HF and lower LF/HF ratio occur correspondingly [61]. According to Burlacu et al., such an application can decrease all-cause rehospitalization and all-cause emergency visits, and mitigate psychological states such as depression or stress [60]. Since HRVB can also stimulate the baroreflex system, it is believed that this intervention could mitigate respiratory diseases, BP, and CVD [60], yet this stance has not been confirmed [59]. Once the commercialization and mass production of portable devices for HRV measurement has commenced, the outcomes of HRVB training can be achieved without geographical limitations, potentially lowering HRV indices and the risk of all-cause death [60].

In addition, physical exercises could also benefit the application of HRV. Studies found that all types of exercise, including endurance, resistance, and combined exercises, improved all the HRV indices, whereas the frequency and duration of exercise did not significantly affect the effectiveness of exercises [54,62]. On the other hand, another study reported that cardiac parasympathetic modulation was potentially lowered after the application of exercise, where resistance exercises had more potential than aerobic exercises, and the employment of exercise is more significant in patients in their mid-adulthood [58].

#### 4.3.2. Risk Stratification

Due to technological advancement, the use of HRV parameters in risk stratification has become more achievable. As mentioned in previous sections, HRV parameters have significant predictive values for diseases like CVD and IDH. Such an indicator is believed to work as a pre-defined criterion for risk stratification, providing an objective index for potential diseases and mortality [63,64]. In research by Gussak, the author found the prognostic significance of non-linear HRV in the risk stratification of patients regarding CV mortality prediction [65]. Clinically, using HRV parameters to preselect patients with a high risk of CVD was found to be more effective than postinfarction risk stratification [66]. Another clinical example of HRV risk stratification can be seen in Perkiömäki’s research [67], where it was concluded that after acute myocardial infarction, a drop in left ventricular function can be predicted in patients with ventricular tachyarrhythmias through HRV risk stratification analysis. However, this potential also has limitations. For the application of risk stratification, the detailed parameters and methods regarding time points, frequency, interdialytic intervals, tested positions, and duration of the observation should be clarified in future studies.

#### 4.3.3. Real-Time HRV Monitoring

The real-time measurement of HRV can solve the issue of difficulty accessing 24 h HRV data, assisting researchers in gaining more accurate information. Recent years have witnessed a mounting number of HRV monitoring devices with the latest attributes, such as being Internet of Things (IoT)-based [68], wearable [69], having sub-Gaussian fitting embedded [70], and having a continuous-wave Doppler radar [71]. These devices help researchers and physicians to understand whether the HRV change points exceed/fall short of the cut-off points and further predict the occurrence of specific diseases [70]. By employing such real-time HRV monitoring methods/devices, interventions could be implemented earlier, before diseases are detected or the patient deteriorates. Such an application is crucial for patients in intensive care unit settings. The predictive values of it can provide helpful information for instant treatments and medical judgment [72]. However, the cut-off points are not defined and united; thus, the data might not be precise.

## 5. Limitations

Although the benefits of HRV have been widely discussed in the previous sections, some limitations exist in the current review, as well as in the knowledge regarding the clinical significance of HRV in dialysis patients. The main limitation is the varied designs of the studies (e.g., time points of HRV measurement, choices of HRV indices, and utility of HRV values), and the number of studies is not abundant enough to perform a more objective review, such as meta-analysis. Although we have enrolled as much of the literature as possible, a narrative review is subject to bias.

## 6. Conclusions

The values and changes of many HRV indices are associated with MetS, malnutrition status, IDH, MACE, mortality, and VAF in ESKD patients. These findings emphasize the vital role of ANS function, including its sympathetic and parasympathetic activities, in responses to external stimuli. A higher level of sympathetic activity at the resting stage and an inadequate elevation of sympathetic activity when facing stress might indicate an unfavorable prognosis among ESKD patients. Despite these shortcomings, HRV might have applications such as HRVB, risk stratification, and real-time HRV monitoring. Further study is encouraged in order to gain a clearer understanding of the clinical significance and application of HRV, and thereby enhance the care of ESKD patients.

## Figures and Tables

**Table 1:** Indices and meanings of three models of HRV assessment.

Meaning of Indices	Time Domain	Frequency Domain	Non-Linear
Autonomic nervous activity	SDNN	TP	SD2
Sympathetic activity	-	LF, LF%, nLF, * LF/HF ratio	-
Parasympathetic activity	RMSSD, NN50 (%)	HF, HF%, nHF, VLF	SD1

Note: ∗ denotes an index of sympathetic to parasympathetic balance. Abbreviations: HF, high frequency; HRV, heart rate variability; LF, low frequency; nHF, normalized high frequency; nLF, normalized low frequency; NN50, number of pairs of adjacent NN intervals differing by more than 50 ms in the entire recording; RMSSD, root mean square successive differences; SD1, standard deviation of instantaneous beat-to-beat interval variability; SD2, continuous long-term R-R interval variability; SDNN, standard deviation of NN intervals; TP, total power; VLF, very low frequency.

**Table 2:** Summary of clinical significance of HRV in dialysis patients.

Findings of HRV and Clinical Outcomes	Population (Number)	Clinical Interpretations	Reference
Metabolic Syndrome			
[5 min ECG; 4 HRV measurements, pre-HD and in early/middle/late phases of HD] - Patients with MetS (+) had significantly lower values of several HRV indices - Patients with the FPG (+) criterion significantly influenced most HRV indices, which were more significant than the influence of MetS	HD patients (*n* = 175)	Higher FPG is associated with decreased autonomic, sympathetic, and parasympathetic activities in HD patients.	Chang et al. (2016) [16]
Malnutrition			
[5 min ECG; 4 HRV measurements, pre-HD and in early/middle/late phases of HD] - Independently lower VLF, TP, Var, and LF% in patients with serum albumin < 3.8 g/dL - Independently higher LF% and LF/HF ratio, but lower HF% in patients with total cholesterol < 100 mg/dL - Independently higher LF% and LF/HF ratio, and lower HF and HF% in patients with body mass index < 23 kg/m^2^ - Independently higher HF% but lower LF% and LF/HF ratio in patients with BW loss (>10% within six months, or >5% within three months) - Independently lower HF% in patients with normalized protein catabolic rate < 1.1 g/kg BW/d	HD patients (*n* = 175)	The malnutrition status defined by the criteria of protein-energy wasting syndrome is associated with ANS dysfunction in HD patients.	Wu et al. (2019) [15]
Mortality			
[5 min ECG; pre-HD HRV] - A higher LF/HF ratio in the pre-HD HRV measurement was an independent predictor of all-cause mortality (150 months of median follow-up)	HD patients (*n* = 41)	A higher sympathetic activity level before HD is associated with a higher mortality risk in HD patients.	Kuo et al. (2018) [36]
[Average of 5 min HRV during the first hour of HD using Holter ECG] - A lower LF/HF ratio during the first hour of HD was an independent predictor for all-cause mortality (54.8 months of median follow-up)	HD patients (*n* = 72)	A lower sympathetic activity level during the first hour of HD is associated with higher mortality in HD patients.	Poulikakos et al. (2018) [37]
[Holter ECG; 3 HRV measurements, pre-HD, during HD, and post-HD] - Post-HD HRV measurement, but not pre-HD or during HD HRV measurements, independently predicted all-cause mortality - Lower VLF, nLF, LF/HF ratio, and higher nHF in post-HD HRV measurement independently predicted all-cause mortality (40 months of median follow-up)	HD patients (*n* = 163)	A lower sympathetic activity level after HD is associated with a higher mortality risk in HD patients.	Osataphan et al. (2023) [6]
[5 min ECG; 2 HRV measurements, pre-HD and post-HD] - ΔLF% higher than the median value was associated with a higher survival rate - Decreased ΔLF% correlated with increased all-cause and cardiovascular mortality - Adding ΔLF%-assisted prediction for all-cause and cardiovascular mortality	HD patients (*n* = 182)	The inability to raise sympathetic activity during HD is associated with a higher mortality risk in HD patients.	Chen et al. (2016) [38]
[5 min ECG; 4 HRV measurements, pre-HD and in early/middle/late phases of HD] - Higher nHF and lower VLF, variance, nLF, and LF/HF ratio were independent predictors for cardiovascular mortality - Higher nHF was an independent predictor for infection-associated mortality (within the follow-up period of 96 months)	HD patients (*n* = 164)	A lower sympathetic activity level during HD is associated with a higher mortality risk in HD patients.	Chang et al. (2020) [7]
[Meta-analysis] - Decreased HRV was associated with higher risks of all-cause mortality and cardiovascular mortality - A long-term ECG recording is better than a short-term recording in predicting all-cause mortality - Decreased SDANN and LF/HF ratio were predictors of all-cause mortality - Decreased SDNN, SDANN, LF/HF ratio, and Ln LF were predictors of cardiovascular mortality	HD patients (*n* = 1175, from 7 studies)	Lower total autonomic nervous activity and sympathetic activity are associated with a higher mortality risk in HD patients.	Yang et al. (2020) [14]
[5-min ECG at 0800–1000 a.m.] - Higher RMSSD, SDSD, and nHF, but lower nLF and LF/HF ratio in non-survivors than survivors - Lower LF/HF ratio was an independent predictor of all-cause mortality (within the follow-up period of 43.8 months)	PD patients (*n* = 81)	A lower sympathetic activity level is associated with a higher mortality rate for PD patients.	Pei et al. (2015) [39]
[24 h Holter ECG] - Decreased DFAα1 was a strong predictor for cardiac and total mortality (34 months of median follow-up)	PD patients (*n* = 132)	Lower sympathetic activity with higher vagal activity is associated with higher cardiac and all-cause mortality in PD patients.	Chiang et al. (2016) [9]
Major adverse cardiovascular events			
[24 h Holter ECG; pre-HD HRV] - Lower SDNN and SDANN were associated with higher MACE development and lower MACE-free survival rates - Lower SDNN was an independent predictor for MACE development (in non-diabetic patients, but not diabetic patients) (32 months of mean follow-up)	HD patients (*n* = 90)	A lower ANS activity level is associated with a higher MACE risk.	Kida et al. (2017) [40]
[5 min ECG, pre-HD HRV] - Kaplan–Meier curves found decreasing MACE-free survival rates in the patients with lower VLF levels - A low VLF activity level was an independent predictor for MACE and hospitalization (33 months of mean follow-up)	HD patients (*n* = 179)	A lower cardiac response toward external stress at the pre-HD stage is associated with a higher MACE risk.	Huang et al. (2017) [21]
[Average of 5 min HRV during the first hour of HD using Holter ECG] - A lower LF/HF ratio during the first hour of HD was an independent predictor for MACE (54.8 months of median follow-up)	HD patients (*n* = 72)	Lower sympathetic activity during the first hour of HD is associated with a higher MACE risk in HD patients	Poulikakos et al. [37]
Intradialytic hypotension			
[5 min ECG; 4 HRV measurements, pre-HD and in early/middle/late phases of HD] - The patients with IDH had statistically lower levels of many HRV indices since the middle phase of HD - By logistic regression analysis, a higher LF/HF ratio and lower variance at HD initiation were independently associated with IDH	HD patients (*n* = 171)	A lower sympathetic activity level at an early stage of HD predicted a higher risk of IDH.	Chang et al. (2016) [22]
[5 min ECG; 3 HRV measurements, in early/middle/late phases of HD] - The changes of many HRV indices between the early and middle phases of HD (ΔHRV) were independent predictors for IDH within the following month. - The ΔHRV variables significantly improved the predictive performance of a multivariate model, and IDH could be predicted by adding to this model	HD patients (*n* = 71)	A minor increase in ANS activity during HD is associated with a higher IDH risk within the coming month.	Park et al. (2019) [41]
[Holter ECG; in non-HD phase, from post-HD to next pre-HD] - VLF and HF were significantly lower in the IDH group - HRV-IDH ≥ 0.544 was an independent factor for IDH (odds ratio 6.14, *p* = 0.11)	HD patients (*n* = 70)	ANS dysfunction determined by the HRV-IDH index is an independent risk factor for IDH.	Park et al. (2024) [42]
Vascular Access Failure			
[5 min ECG; 4 HRV measurements, pre-HD and in early/middle/late phases of HD] - A lower LF/HF ratio and higher nHF occurred in patients with VAF than those without VAF - LF/HF ratio and nHF were two independent indicators for VAF	HD patients (*n* = 175)	Lower and higher sympathetic activity is associated with VAF in the arteriovenous fistula and Hickman’s catheter, respectively.	Huang et al. (2017) [19]

Abbreviations: ANS, autonomic nervous system; BW, body weight; DFAα1, detrended fluctuation analysis alpha 1; ECG, electrocardiography; FPG, fasting plasma glucose; HD, hemodialysis; HF, high frequency; HF%, high frequency percentage; HRV, heart rate variability; IDH, intradialytic hypotension; LF, low frequency; LF/HF ratio, low-to-high frequency ratio; LF%, low frequency percentage; MACE, major adverse cardiovascular events; MetS, metabolic syndrome; nHF, normalized HF; nLF, normalized LF; PD, peritoneal dialysis; RMSSD, root mean square successive differences; SDANN, standard deviation of the average NN intervals; SDNN, standard deviation of normal-to-normal interval; SDSD, standard deviation of the differences between successive NN intervals; TP, total power; VAF, vascular access failure; VLF, very low frequency.

**Table 3:** Associations between HRV indices and patients’ outcomes.

HRV Indices	Mortality	MACE	IDH	VAF
nLF	Post-HD nLF↓, mortality↑ [6] During HD nLF↓, CV mortality↑ [7]	-	-	-
ΔLF%	ΔLF% > 5.1 nu, mortality↓ ΔLF%↓, mortality↑ [38]	-	-	-
VLF	Post-HD VLF↓, mortality↑ [6] Pre-HD VLF↓, CV mortality↑ [7]	VLF↓, MACE↑ [6]	Post-HD VLF↓, IDH↑ [42]	-
HF	Post-HD HF↑, HD mortality↑ [6]	-	Post-HD VLF↓, HF↑ [42]	-
nHF	post-HD nHF↑, mortality↑ [6] During HD nHF↑, CV mortality↑ [7] During HD nHF↑, infection mortality↑ [7]	-	-	During HD nHF↑, VAF↑ [19]
LF/HF ratio	Pre-HD LF/HF ratio↑, mortality↑ [36] Post-HD LF/HF ratio↓, mortality↑ [6] During HD LF/HF ratio↓, CV mortality↑ [7] During HD LF/HF ratio↓, mortality↑ [37]	During HD LF/HF ratio↓, MACE↑ [37]	LF/HF↑, IDH↑ at the beginning of HD [22]	LF/HF↓, VAF↑ [19]
SDNN	-	SDNN↓, MACE↑ [40]	-	-
Variance	Variance↓, CV mortality↑ [7]	-	Variance↓, IDH↑ at the beginning of HD [22]	-

Note: “↑” and “↓” stand for increase and decrease, respectively. “-” means not applicable. Abbreviations: CV, cardiovascular; HD, hemodialysis; HF, high frequency; HRV, heart rate variability; IDH, intradialytic hypotension; LF, low frequency; MACE, major adverse cardiovascular events; nHF, normalized high frequency; nLF, normalized high frequency; SDNN, standard deviation of the average NN intervals; VAF, vascular access failure; VLF, very low frequency.

## Data Availability

Not applicable.
